# Industry Sectors Highly Affected by Worksite Outbreaks of Coronavirus Disease, Los Angeles County, California, USA, March 19–September 30, 2020

**DOI:** 10.3201/eid2707.210425

**Published:** 2021-07

**Authors:** Zuelma Contreras, Van Ngo, Marifi Pulido, Faith Washburn, Gayane Meschyan, Fruma Gluck, Karen Kuguru, Roshan Reporter, Condessa Curley, Rachel Civen, Dawn Terashita, Sharon Balter, Umme-Aiman Halai

**Affiliations:** Los Angeles County Department of Public Health, Los Angeles, California, USA

**Keywords:** coronavirus disease, COVID-19, severe acute respiratory syndrome coronavirus 2, SARS-CoV-2, respiratory infections, workplace, incidence, industry sectors, manufacturing¸ retail trade, transportation and warehousing, outbreaks, public health, food safety, zoonoses, Los Angeles County, United States

## Abstract

Worksites with on-site operations have experienced coronavirus disease (COVID-19) outbreaks. We analyzed data for 698 nonresidential, nonhealthcare worksite COVID-19 outbreaks investigated in Los Angeles County, California, USA, during March 19, 2020‒September 30, 2020, by using North American Industry Classification System sectors and subsectors. Nearly 60% of these outbreaks occurred in 3 sectors: manufacturing (n = 184, 26.4%), retail trade (n = 137, 19.6%), and transportation and warehousing (n = 73, 10.5%). The largest number of outbreaks and largest number and highest incidence rate of outbreak-associated cases occurred in manufacturing. Furthermore, 7 of the 10 industry subsectors with the highest incidence rates were within manufacturing. Approximately 70% of outbreak-associated case-patients reported Hispanic ethnicity. Facilities employing more on-site staff had larger and longer outbreaks. Identification of highly affected industry sectors and subsectors is necessary for targeted public health planning, outreach, and response, including ensuring vaccine access, to reduce burden of COVID-19 in vulnerable workers.

Worksites that have had on-site operations during the coronavirus disease (COVID-19) pandemic have been vulnerable to COVID-19 outbreaks. The effect of COVID-19 on essential workers in food manufacturing has been well-described, but limited data exist on the burden of COVID-19 in other industry sectors (*1*). The high-density, fast-paced environments of food production facilities pose a barrier to proper adherence to COVID-19 prevention measures, such as social distancing, use of face coverings, and cleaning of shared spaces (*2*). These challenges are not unique to food production facilities. Furthermore, factors distinctive to other sectors, such as increased contact with the public, could similarly increase the risk of COVID-19 worksite exposure. A closer examination of the COVID-19 burden in multiple industry sectors, particularly within their specific subsectors, is warranted to provide a more complete characterization of the risk and impact of COVID-19 exposure in worksites.

In Los Angeles County, California, USA, the first COVID-19 worksite outbreak was identified by the Los Angeles County Department of Public Health (LACDPH; Los Angeles, CA, USA) on March 19, 2020, and by September 30, 2020, LACDPH had investigated 698 worksite outbreaks. Safer at home orders required all nonessential businesses in Los Angeles County to close operations during March 16‒May 8, 2020, when some businesses opened under modified operations. The number of COVID-19 worksite outbreaks mirrored trends in community transmission. Worksite outbreak numbers increased until mid-July, followed by a gradual decrease until September 30. This analysis identifies the industries that were most affected by COVID-19 outbreaks in Los Angeles County during March 19‒September 30, 2020, and describes worksite outbreak characteristics to understand the risk of exposure at the various worksites and to help guide public health outbreak prevention and response strategies.

## Methods

### Outbreak Identification

This analysis included COVID-19 outbreaks at nonresidential worksites in Los Angeles County but excluded healthcare settings, homelessness services, and emergency medical services because outbreaks in these settings are investigated under different public health protocols. We excluded outbreaks in Pasadena and Long Beach because they have their own health departments. Initially, a worksite outbreak was identified when >5 suspected or laboratory-confirmed COVID-19 cases occurred within 14 days, with >1 case being laboratory-confirmed. On May 29, upon increased testing capacity and development of state definitions, a worksite outbreak was subsequently defined as >3 laboratory-confirmed cases occurring within 14 days. A county health officer order was issued requiring worksites to report any suspected outbreaks that might meet the definition. All reported, suspected outbreaks were investigated by LACDPH to determine if the cluster met outbreak criteria, including presence of epidemiologic links between cases indicating worksite transmission. Persons with COVID-19 were determined to be outbreak-associated cases on the basis of timing of symptoms or positive test result, exposure at the worksite during the investigation period, and absence of verifiable COVID-19 exposure outside the worksite.

### Outbreak Investigation Procedures

Worksite outbreaks were investigated by an investigation team consisting of a public health investigator or public health nurse, a physician, and an environmental health inspector. Guidance on COVID-19 best practices was issued by the outbreak investigator to the worksite; guidance included recommendations on isolation of cases, contact investigation in the workplace, testing of close contacts, entry screening, physical distancing, masking, and cleaning/disinfection protocols. In addition, we conducted telephone conferences, as well as on-site visits, if needed, to assess worksite compliance with COVID-19 safety protocols. Worksites that failed to comply risked closure. Worksites were required to submit case line lists to LACDPH, and these lists were used for documentation and tracking of outbreak-associated cases at each site. The public health investigator regularly communicated with the site contact during each worksite outbreak (3‒5 times/wk) to monitor for additional cases until at least 2 weeks after the last outbreak-associated case.

### Analysis of Outbreak Data

We classified outbreaks by industry sector and subsector as described by the North American Industry Classification System used for classifying businesses (*3*). We calculated the outbreak-associated case incidence rate (IR) per 100,000 persons by using average annual employment data from the 2019 Quarterly Census of Employment and Wages (QCEW) for Los Angeles County (*4*). Because IR denominators include only employees, we excluded cases in nonemployees from IR calculations. We calculated outbreak duration by using symptom onset or test date, whichever was earlier, of the first and last outbreak-associated case. We calculated the Spearman correlation coefficient (ρ) to assess the strength and direction of association between the number of staff and number of outbreak-associated cases, as well as outbreak duration. We used descriptive statistics to summarize data. χ^2^ tests were used to assess differences in case characteristics between sectors. All analyses were conducted by using SAS version 9.4 (https://www.sas.com).

## Results

### Worksite Outbreaks by Industry Sector

This analysis included 698 worksite outbreaks identified by LACDPH during March 19‒September 30, 2020, of which 14% (n = 96) were still under investigation at the time of analysis. A total of 7,625 cases were associated with these outbreaks. We provide descriptive statistics for worksite outbreaks by North American Industry Classification System sector (Table 1). Most outbreaks occurred in manufacturing (n = 184, 26.4%), retail trade (n = 137, 19.6%), and transportation and warehousing (n = 73, 10.5%). Outbreak-associated cases were highest in manufacturing (n = 3,319, 43.5%), transportation and warehousing (n = 980, 12.9%), and retail trade (n = 871, 11.4%). 

**Table 1:** Descriptive statistics for worksite outbreaks of coronavirus disease, by North American Industry Classification System industry sector, Los Angeles County, California, USA, March 19–September 30, 2020*

Sector	No. (%) outbreaks	No. (%) outbreak-associated cases	Average no. employed annually†	Outbreak-associated incidence‡	Median duration of outbreaks, d (min–max)	Median no. outbreak-associated cases (min–max)	Median no. staff at outbreak sites (min–max)
Overall total	698 (100.0)	7,625 (100.0)	4,439,578	171.8	13.0 (0–189)	6.0 (3–277)	95.0 (3–8,585)
Accommodation and food services	71 (10.2)	346 (4.5)	448,709	77.1	9.0 (0–71)	4.0 (3–16)	29.0 (3–180)
Administrative and support and waste management and remediation services	14 (2.0)	100 (1.3)	278,535	35.9	11.0 (1–85)	6.0 (3–17)	40.0 (11–239)
Arts, entertainment, and recreation	3 (0.4)	63 (0.8)	107,967	58.4	41.0 (13–62)	22.0 (3–38)	302.0 (134–1,500)
Construction	27 (3.9)	257 (3.4)	149,695	171.7	7.0 (1–83)	6.0 (3–81)	50.0 (7–3,000)
Educational services	11 (1.6)	62 (0.8)	380,928	12.3	7.0 (0–53)	5.0 (3–14)	69.5 (22–249)
Finance and insurance	9 (1.3)	66 (0.9)	134,635	49.0	11.0 (1–30)	4.0 (3–22)	18.0 (4–201)
Healthcare and social assistance§	29 (4.2)	199 (2.6)	777,828	20.7	11.0 (0–35)	6.0 (3–27)	68.0 (10–347)
Information	10 (1.4)	46 (0.6)	210,439	21.9	7.0 (1–23)	4.0 (3–9)	58.5 (20–140)
Manufacturing	184 (26.4)	3,319 (43.5)	338,308	980.8	20.0 (3–189)	9.0 (3–277)	153.5 (5–7,000)
Mining, quarrying, and oil and gas extraction	1 (0.1)	3 (0.0)	1,895	158.3	9.0 (9–9)	3.0 (3–3)	22.0 (22–22)
Other services (except public administration)	10 (1.4)	66 (0.9)	154,961	42.6	11.0 (2–36)	6.0 (3–13)	31.0 (8–120)
Professional, scientific, and technical services	10 (1.4)	50 (0.7)	299,007	16.7	6.5 (1–21)	4.0 (3–16)	20.0 (3–216)
Public administration	44 (6.3)	483 (6.3)	174,522	276.2	12.0 (2–117)	6.0 (3–67)	160.0 (6–1,200)
Real estate and rental and leasing	8 (1.2)	36 (0.5)	88,646	38.4	7.5 (0–11)	5.0 (3–7)	22.0 (6–115)
Retail trade	137 (19.6)	871 (11.4)	416,640	208.3	12.0 (0–141)	5.0 (3–25)	99.0 (5–8,585)
Transportation and warehousing	73 (10.5)	980 (12.9)	230,039	425.1	23.0 (0–158)	9.0 (3–125)	255.0 (4–2,083)
Utilities	3 (0.4)	14 (0.2)	28,370	49.3	10.0 (5–11)	3.0 (3–8)	19.0 (10–71)
Wholesale trade	54 (7.7)	664 (8.7)	218,454	304.0	18.0 0–79)	8.0 (3–87)	84.0 (9–600)

*Max, maximum; min, minimum. †Denominator data were derived from 2019 Quarterly Census of Employment and Wages for Los Angeles County. ‡Per 100,000 persons. Incidence rate calculations excluded cases in nonemployees (n = 62). §Full name of sector is healthcare and social assistance, but analysis includes only worksites in social assistance.

A total of 62 cases in nonemployees were excluded from IR calculations. Most (n = 15) nonemployee cases were in students within the educational services sector and children in daycare (n = 38) within the healthcare and social assistance sector. The remaining 9 nonemployee cases were spread across multiple different sectors and identified as being in vendors/contractors working on-site during the outbreak. The overall outbreak-associated IR was 171.8. The highest IRs were for manufacturing (980.8), transportation and warehousing (425.1), and wholesale trade (304.0). The overall median cases per outbreak was 6 (range 3‒277), median on-site staff per outbreak was 95 (range 3‒8,585), and median outbreak duration was 13 (range 0‒189) days. The number of on-site employees showed a moderately positive correlation with the number of outbreak-associated cases (ρ = 0.49), as well as outbreak duration (ρ = 0.54) (p<0.05).

### Outbreak-Associated Case-Patient Characteristics

Of 7,625 outbreak-associated case-patients, 79% (n = 6,047) were ≥18 years of age and had demographic and outcome information available for analysis. Outbreak-associated case-patients were predominantly ≤50 years of age, male, and Hispanic; there were some differences by sector (p<0.05) (Table 2). The other services sector, comprised primarily of repair and maintenance businesses, was the only sector in which most (55.7%) case-patients were ≥50 years of age. The sectors that had <50% male case-patients were healthcare and social assistance (22.1%); finance and insurance (35.7%); and professional, scientific, and technical services (44.4%). The proportion of Hispanic persons was highest in manufacturing (78.9%), followed by accommodation and food services (72.3%) and arts, entertainment, and recreation (71.4%). A few sectors had a lower proportion of cases in Hispanic persons than in non-Hispanic persons: educational services (37.0%); professional, scientific, and technical services (46.7%); and public administration (38.7%). A total of 243 hospitalizations (4%) and 37 deaths (0.6%) occurred; no differences were observed by sector or race/ethnicity (p>0.05).

**Table 2:** Coronavirus disease outbreak‒associated case demographics and health outcomes, by North American Industry Classification System industry sectors, Los Angeles County, California, USA, March 19–September 30, 2020*

Sector	Male sex†	Age ≥50 y†	Hispanic†	Hospitalizations	Deaths
Overall total	3,570/5,929 (60.2)	1,773/6,047 (29.3)	2,511/3,567 (70.4)	243/6,047 (4.0)	37/6,047 (0.6)
Accommodation and food services	135/263 (51.3)	62/267 (23.2)	99/137 (72.3)	9/267 (3.4)	1/267 (0.4)
Administrative and support and waste management and remediation services	58/89 (65.2)	30/89 (33.7)	29/43 (67.4)	5/89 (5.6)	0/89 (0.0)
Arts, entertainment, and recreation	16/26 (61.5)	13/26 (50.0)	15/21 (71.4)	4/26 (15.4)	1/26 (3.8)
Construction	156/160 (97.5)	36/167 (21.6)	57/92 (62.0)	4/167 (2.4)	0/167 (0.0)
Educational services	29/55 (52.7)	15/55 (27.3)	10/27 (37.0)	2/55 (3.6)	0/55 (0.0)
Finance and insurance	20/56 (35.7)	20/56 (35.7)	21/33 (63.6)	5/56 (8.9)	0/56 (0.0)
Health care and social assistance§	31/140 (22.1)	42/143 (29.4)	47/93 (50.5)	7/143 (4.9)	2/143 (1.4)
Information	21/33 (63.6)	9/33 (27.3)	8/13 (61.5)	0/33 (0.0)	0/33 (0.0)
Manufacturing	1,514/2,689 (56.3)	1,002/2,754 (36.4)	1,325/1,680 (78.9)	138/2,754 (5.0)	25/2,754 (0.9)
Mining, quarrying, and oil and gas extraction	1/1 (100.0)	1/1 (100.0)	0/0 (0.0)	0/1 (0.0)	0/1 (0.0)
Other services (except public administration)	45/61 (73.8)	34 (55.7)	26/37 (70.3)	1/61 (1.6)	0/61 (0.0)
Professional, scientific, and technical services	20/45 (44.4)	10/46 (21.7)	14/30 (46.7)	0/46 (0.0)	0/46 (0.0)
Public administration	208/292 (71.2)	54/294 (18.4)	74/191 (38.7)	9/294 (3.1)	2/294 (0.7)
Real estate and rental and leasing	21/32 (65.6)	10/32 (31.3)	12/19 (63.2)	0/32 (0.0)	0/32 (0.0)
Retail trade	377/656 (57.5)	131/676 (19.4)	262/394 (66.5)	19/676 (2.8)	1/676 (0.1)
Transportation and warehousing	498/787 (63.3)	153/796 (19.2)	326/483 (67.5)	25/796 (3.1)	3/796 (0.4)
Utilities	8/12 (66.7)	2/13 (15.4)	6/9 (66.7)	1/13 (7.7)	0/13 (0.0)
Wholesale trade	412/532 (77.4)	149/538 (27.7)	180/265 (67.9)	14/538 (2.6)	2/538 (0.4)

*Values are no. in category/total no. (%). †p<0.05 by χ^2^ test. ‡Full name of sector is health care and social assistance, but analysis includes only worksites in social assistance.

### Worksite Outbreaks by Industry Subsector

We further analyzed worksite outbreaks by industry subsectors. Among the 69 subsectors represented in our data, most outbreaks were in food and beverage stores (n = 75, 10.7%; sector: retail trade), followed by food manufacturing (n = 70, 10.0%; sector: manufacturing) and food services and drinking places (n = 64, 9.2%; sector: accommodation and food services). The highest number of outbreak-associated cases among subsectors were in food manufacturing (n = 1,515, 19.9%; sector: manufacturing); warehousing and storage (n = 621, 8.8%; sector: transportation and warehousing); and apparel manufacturing (n = 595, 7.8%; sector: manufacturing). Subsectors within the manufacturing, transportation and warehousing, and public administration sectors had the highest IRs (Table 3). The top 3 subsectors by IR were food manufacturing (3,779.2), warehousing and storage (2,853.2), and apparel manufacturing (2,185.7).

**Table 3:** Descriptive statistics for 10 North American Industry Classification System industry subsector that had the highest outbreak-associated incidence rates for coronavirus disease, Los Angeles County, California, USA, March 19–September 30, 2020*

Subsector	Sector	No. (%) outbreaks	No. (%) outbreak-associated cases	Average no. employed annually†	Outbreak-associated incidence‡	Median no. outbreak-associated cases (min–max)
Food manufacturing	Manufacturing	71 (10.2)	1,592 (20.9)	40,088	3,971.3	11.0 (3–277)
Warehousing and storage	Transportation and Warehousing	35 (5.0)	621 (8.1)	21,765	2,853.2	10.0 (3–125)
Apparel manufacturing	Manufacturing	15 (2.1)	595 (7.8)	27,223	2,185.7	16.0 (3–184)
Beverage and tobacco product manufacturing	Manufacturing	6 (0.9)	99 (1.3)	6,357	1,557.3	10.5 (5–50)
Electrical equipment, appliance, and component manufacturing	Manufacturing	7 (1.0)	130 (1.7)	8,694	1,495.3	7.0 (3–68)
Plastics and rubber products manufacturing	Manufacturing	10 (1.4)	92 (1.2)	11,476	801.7	7.5 (3–22)
Furniture and related product manufacturing	Manufacturing	11 (1.6)	97 (1.3)	12,263	791.0	7.0 (4–24)
Chemical manufacturing	Manufacturing	9 (1.3)	141 (1.8)	19,656	717.3	8.0 (3–58)
Couriers and messengers	Transportation and Warehousing	14 (2.0)	213 (2.8)	32,195	655.4	16.0 (5–31)
Justice, public order, and safety activities	Public Administration	37 (5.3)	443 (5.8)	72,265	611.6	6.0 (3–67)

*Only rates for subsectors with >20 cases are included. Max, maximum; min, minimum. †Denominator data were derived from 2019 Quarterly Census of Employment and Wages for Los Angeles County. ‡Per 100,000 persons. Incidence rate calculations excluded cases in nonemployees (n = 62).

## Discussion

The manufacturing, transportation and warehousing, and retail trade sectors had the highest number of COVID-19 outbreaks and outbreak-associated cases among 698 worksite outbreaks in Los Angeles County. Manufacturing had the highest IR, which was >5 times the overall IR and twice that of the next highest sector. Among the top 10 subsectors by IR, 7 were from the manufacturing sector. Many worksites within the most affected subsectors were among those designated as essential critical infrastructure in California, enabling continued on-site operations through the pandemic. In addition, some nonessential manufacturing worksites redirected operations to the production of essential goods. Continued in-person operations probably contributed to increased risk of COVID-19 exposure and transmission at these facilities. Four of 5 outbreak-associated case-patients within manufacturing were Hispanic, the highest number for any sector. Worksite outbreak data can help identify vulnerable workers and enable public health departments to target policies and response, including ensuring vaccine access, to employees most affected by COVID-19.

These findings are supported by an analysis in Utah that reported similar results in manufacturing (*5*). In contrast, construction was not a highly affected sector in Los Angeles County on the basis of number of outbreaks, outbreak-associated cases, or IR. Jurisdictional differences in affected industries might vary by workforce distribution, reporting practices, and local outbreak identification and investigation procedures. This analysis identified affected subsectors, which might be essential for public health departments planning in diverse sectors (e.g., manufacturing) that require subsector-specific considerations. The food manufacturing subsector had the highest IR among subsectors in our analysis and is known to be a high-risk industry (*1*). This study identified additional manufacturing subsectors, such as apparel manufacturing and electrical equipment, appliance, and component manufacturing, which had among the highest IRs.

Facilities with more on-site staff are at risk for larger and longer COVID-19 outbreaks and should develop and implement strict safety protocols to prevent worksite exposure and transmission. In addition to having the most outbreak-associated cases, manufacturing and transportation and warehousing had among the most on-site employees and longest outbreak durations. The high-density environments and close contact in production lines, long shifts, shared equipment, and common spaces might increase risk for exposure in manufacturing and warehousing settings (*6*). In addition, practices such as use of shared transportation and frequent off-site worker interaction might contribute to this risk (*6*). Poor ventilation and sanitation have been well-documented in apparel manufacturing (*7*). Worksites within retail trade also had a high burden of COVID-19 outbreaks. Food and beverage stores had the most outbreaks within retail trade. Workers in these settings are particularly at risk for COVID-19 exposure because of their increased contact with the public (*6*).

Differences in worksite compliance with COVID-19 prevention protocols could also account for the higher COVID-19 burden seen in some industries. LACDPH site inspections, conducted in response to public complaints, have noted lower compliance with COVID-19 reopening and safety protocols in apparel manufacturing compared to restaurants (subsector: food services and drinking places) and grocery stores (subsector: food and beverage stores). Recommendations such as physical distancing might be more challenging to implement in manufacturing sites because of interdependent workflow processes and less modifiable physical environments. In addition, food facilities such as grocery stores and restaurants that routinely interact with public health departments because of permit requirements or regular inspections might have more knowledge and experience responding to DPH recommendations, which could contribute to higher compliance in these settings. Limited data has been published on compliance with COVID-19 prevention measures and potential barriers to compliance by industry. A closer and more systematic analysis of compliance with infection control measures by industry sector/subsector is needed.

Hispanic persons comprised 70% of outbreak-associated case-patients, which is almost twice the proportion of Hispanic persons employed in Los Angeles County in the 18 industry sectors represented in this analysis (40%) (*8*). This finding is consistent with findings of previous studies (*1*,*5*). Racial/ethnic minorities are overrepresented within essential industries, which often have higher risk working conditions as described above. In addition, Hispanic persons might experience more language barriers and are less likely to have access to paid leave and flexible work schedules (*9*,*10*). Community case rates of COVID-19 in Los Angeles County by race/ethnicity reflect an overall disproportionate burden on Hispanic persons, and the daily IR for Hispanic persons is more than twice that for white residents (*11*). Regardless of whether workplace exposure has driven community transmission or vice versa, a controlled worksite environment provides an opportunity to mitigate transmission within highly affected communities.

One limitation of our study is that the analysis includes only outbreaks reported to LACDPH, which underestimates the actual number of outbreaks. Because 14% of the investigations were ongoing, some outbreak-associated cases might not yet be documented. Employers might not have knowledge of employee symptom status, health outcomes, and testing results such that cases, outbreaks, hospitalizations, and deaths would remain unknown and unreported. Worksites that conducted facility-wide testing voluntarily or based on LACDPH recommendations probably identified a higher number of cases. Of the 6,047 outbreak-associated cases that had demographic and outcome information available for analysis, 41% were missing race/ethnicity data. However, missing data are assumed to be random across industries.

Outbreak-associated cases represent a fraction of cases in employees that might have occurred in each sector/subsector. Although outbreak-associated case-patients are more likely to have been exposed at the worksite, some non-worksite acquired cases were probably included.

The IR might be underestimated because of inclusion of persons in the denominator who were not captured in the numerator if they became case-patients. Residents of Pasadena and Long Beach were included in the QCEW IR denominator for Los Angeles County, but outbreaks in these cities were excluded. Denominator data were based on 2019 QCEW average annual employment data, which was probably higher than employment in 2020 during the pandemic. However, this difference was probably less pronounced in sectors such as manufacturing that are composed of mostly essential businesses that continued operations. In addition, outbreaks in healthcare settings, homelessness services, and emergency medical services were excluded, underestimating the risks in the health care and social assistance and public administration sectors the most. Finally, the IR might also have been affected by persons who were not captured in the denominator (e.g., QCEW does not capture informal employment, which is more common in certain sectors).

This study highlights key sectors that have been affected by COVID-19 outbreaks and would benefit most from public health outreach and education. A better understanding of employer- and employee-level barriers that decrease compliance with public health measures and directives in specific industries is needed. COVID-19 safety protocols tailored to each industry that are culturally and linguistically appropriate to the employees at the worksite must be developed. Local champions can help build trust and support communication efforts.

Public health departments should cultivate and maintain relationships with labor representatives, worker advocates, and trade associations so that they can remain engaged with public health priorities and can help implement health directives when needed. Public health departments must continue to target essential workers in the affected industries in vaccination efforts to address gaps in vaccine access and barriers to uptake. The burden of disease, as well as the highest ethnic minority representation within manufacturing, underscores this sector as a priority area in Los Angeles County. The COVID-19 pandemic has highlighted infrastructure disparities and labor challenges faced by the Los Angeles County workforce and is an opportunity to improve worker safety and well-being across all industries.
